# Sanitary Aspects of Countering the Spread of COVID-19 in Russia

**DOI:** 10.3390/ijerph182312456

**Published:** 2021-11-26

**Authors:** Elena Belova, Ekaterina Shashina, Denis Shcherbakov, Yury Zhernov, Vitaly Sukhov, Nadezhda Zabroda, Valentina Makarova, Tatiana Isiutina-Fedotkova, Svetlana Mishina, Anton Simanovsky, Oleg Mitrokhin

**Affiliations:** Department of General Hygiene, I.M. Sechenov First Moscow State Medical University (Sechenov University), Trubetskaya St. bldg. 8/2, 119435 Moscow, Russia; shashina_e_a@staff.sechenov.ru (E.S.); shcherbakov_d_v@staff.sechenov.ru (D.S.); zhernov_yu_v@staff.sechenov.ru (Y.Z.); sukhov_v_a@staff.sechenov.ru (V.S.); zabroda_n_n@staff.sechenov.ru (N.Z.); makarova_v_v@staff.sechenov.ru (V.M.); isiutina-fedotkova_t_s@staff.sechenov.ru (T.I.-F.); mishina_s_a@staff.sechenov.ru (S.M.); simanovsky_a_a@staff.sechenov.ru (A.S.); mitrokhin_o_v@staff.sechenov.ru (O.M.)

**Keywords:** COVID-19, public health, risk assessment, governance

## Abstract

Due to the conditions that cause the spread of COVID-19, national health systems worldwide are under severe strain. Most countries face similar difficulties such as a lack of medical personnel and equipment and tools for diagnosis and treatment, overrun hospitals, and forced restriction of planned medical care. Public authorities in healthcare take the following measures due to increased pressure: limiting the transmission and spread of the virus (social distancing and quarantine), mobilizing medical personnel, ensuring the availability of diagnostic and treatment tools, and providing a sufficient number of premises, which are not always suitable for the provision of medical care (buildings and structures). To date, the stages of management decision-making to counter coronavirus infection and the risk of COVID-19 transmission at various facilities have not been analyzed. The authors propose a methodology for assessing the COVID-19 transmission risk at various social and transport facilities. A survey of 1325 respondents from Moscow demonstrated the most significant risk factors, such as visitation avoidance, infection risk, and facemask wearing. Risk categories were determined and objects classified according to high, medium, and low-risk levels.

## 1. Introduction

Outbreaks of infectious diseases, which reach large numbers of people and cause enormous damage to the world economy, are becoming a new reality. Since the end of 2019, humanity has faced a new biological threat in COVID-19, which causes several clinical manifestations—from mild forms of acute respiratory infection to severe acute respiratory syndrome (SARS)—associated with a long period of rehabilitation [1,2].

The spread of COVID-19, which has infected millions of people, and the high mortality rate (hundreds of thousands of people) have shown that public health is not ready to provide mass medical care to patients in pandemic conditions [3,4,5]. In addition, during the period of complete restriction of people’s movement and partial limits on social contacts, economic activity was almost completely stopped, and nearly half of the world’s population was in isolation [6,7], which has become one of the significant factors of the enormous economic damage, a revision of the paradigm of the development of public relations [8,9].

The emergence of COVID-19 has made apparent the threat of epidemics and pandemics for the foreseeable future [10]. Recent studies show that the pandemic crisis can be overcome through state preparedness and planning policies [11,12,13]. Reliable indicators of a country’s preparedness for epidemics at the national level are crucial for assessing global resilience to epidemics and pandemics [14]. Epidemic preparedness reflects the ability of the public health, treatment, and prevention network and government agencies to detect, report, and respond to outbreaks to reduce impacts on public health, society, and the economy [15,16].

The emergence of COVID-19 caused the need to develop new ways to diagnose quickly, provide medical care to the population, and review measures to counter its spread [17,18].

Although there have been significant investments in the global epidemiological surveillance of population health and increased healthcare potential, a large part of the world has shown unpreparedness for infectious disease threats [19,20,21].

In the context of the rapid development of healthcare epidemic processes, a very significant problem arises: the need to rapidly increase bed space for patients with infectious diseases [22]. In addition, the quality and coverage of transport and communication infrastructure can influence the effectiveness of anti-epidemic and preventive measures and the speed of public health responses, ensuring (or limiting) the movement of personnel and the use of quality medical products, including personal protective equipment (PPE), face masks, and gloves [23].

Proposals have been developed to comply with strict infection control measures in medical organizations and the use of PPE in public places to prevent the spread of infection [24,25]. Including infectious disease prevention in the training of medical workers and the education of the population and qualification requirements for the certification of medical workers is justified. Further, including infectious disease prevention issues in training programs for medical workers is substantiated [26].

In the “Great Barrington Declaration” adopted on 4 October 2020, specialists in the field of epidemiology, infectious diseases, and public health identified the need to protect the most vulnerable groups of the population, especially those over 60 years old, and involve specialists in medical organizations with antibodies to COVID-19.

This study aims to analyze regional organizational management decisions aimed at countering the spread of COVID-19 in public health [27].

## 2. Materials and Methods

A survey was conducted, questioning people living in Moscow, to determine the frequency of public place attendance and compliance with the main measures for non-specific prophylaxis of COVID-19 (*n* = 1325).

The questionnaire, developed by the Department of General Hygiene of Sechenov University staff, contained questions to identify the criteria for the most significant risks of contracting COVID-19 among the population at various social facilities. A risk-based approach was used as the methodological basis for identifying objects that increased the risk of spreading COVID-19.

Statistical processing of the research results was carried out using the statistical software package STATISTICA Base. The statistical study of the relationship between the features was carried out using the Spearman nonparametric correlation coefficient (r) with the Fisher transform (z) to approximate the exact distribution of the correlation coefficient. Cluster analysis was used to group the respondents’ answers and highlight informative features to further develop the risk category scale. We applied factor analysis by the method of principal components (at the level >0.70) to those questions on the questionnaire that demonstrated the largest number of statistically significant indicators of Spearman’s correlation coefficient. The total percentage of variance is 60.43%.

A *p*-value less than 0.01 was statistically significant.

## 3. Results

Spearman’s correlation analysis showed that out of 51 questions, 20 demonstrated the largest number of statistically significant indicators of the correlation coefficient. For 20 features, factor analysis was applied by the method of principal components Factor Loadings (Varimax normalized) Extraction: Principal components (Marked loadings are >0.70), which made it possible to distinguish the following three factors:

**Factor 1** is the most informative (28.12%). Its composition is determined by the values of the variables’ positive signs in answer to the question “What are you doing to protect yourself from the coronavirus?” This factor can be identified as the “Behavior Strategy” factor. During the pandemic, these respondents used social distancing and avoided visiting clinics, grocery stores, street outlets and kiosks, and shops selling industrial and household goods.

**Factor 2** has an informative value of 23.44% and is represented by a positive pole of responses from respondents who indicated objects in the urban environment that increase the risk of COVID-19 infection: public land transport, commuter trains (electric trains), pharmacies, and non-food stores. This factor can be identified as the “Risk of infection” factor. Most of the respondents considered these particular objects in the urban environment in their choice, associating them with a high level of COVID-19 infection.

**Factor 3** has an informative value of 8.87% and includes only the positive pole of the mask compliance variables. Respondents cited wearing a mask on public transportation, at workplaces, and when shopping and at a pharmacy as more essential to protect themselves from COVID-19 infection. This factor can be identified as the “Mask Mode” factor.

Hierarchical cluster analysis, carried out to group the respondents’ answers, made it possible to group indicators that can later be used to divide the population of respondents according to the measured characteristics and check the differences further, including in relation to the risk of COVID-19 infection.

The choice of features was determined by the single-link method according to the sequential agglomeration table, which made it possible to trace the dynamics of increasing differences in clustering steps and determine the stage at which a sharp increase in differences is noted. Of the 46 features, 16 were selected. The following data were obtained when calculating the hierarchy of informative features (the hierarchy coefficient equal to 0.7 was determined as the threshold for choosing the leading factors for clustering) (Table 1).

Thus, we have established the most significant risk criteria, which are indicators characterizing visits to various social facilities and trips (and duration) by multiple types of public transport, observance of the mask regime, and social distancing for various objects (objects of risk). The assessment was carried out by awarding points according to gradations of informative signs in the respondents’ answers (Table 2).

Classifying a respondent as a risk category is necessary to assess, in points, each informative feature—a sanitary criterion (Table 3). The average identification value (interval) and higher, obtained from the respondent, requires closer attention.

## 4. Discussion

As of 16 November 2020, over 50 million people in the world had tested positive for COVID-19 [28].

Given this fact, deploying beds in infectious hospitals or reprofiling general facilities as infectious hospitals for all those in need of hospital treatment is impossible. Even in the future, assuming the construction of new infectious hospitals in an epidemic, economic feasibility issues will arise since specialized bed-spaces will be idle when people do not need treatment.

An analysis of COVID-19 regulatory documents, including in medical organizations, has shown that the first recommendations, instructions, and decisions of the Russian Federal State Agency for Health and Consumer Rights (Rospotrebnadzor) were published from 21 to 25 January 2020. By the end of January 2020, testing systems for SARS-Cov-2 detection were developed, and their production had begun. The National Plan for the Prevention of the Import and Spread of COVID-19 was approved [29].

The power to abolish (prolong) temporary restrictions imposed in connection with the spread of COVID-19 from May 2020 were transferred to the governors (heads of regions). At the federal level, the following tasks were solved:

- Protection of the country’s external borders from citizens with COVID-19 arriving from abroad and air and rail services with foreign countries were almost completely stopped (except for charter flights for citizens who expressed a desire to return to Russia);
- Social and economic support of the population, creation of temporary jobs, and support of scientific, educational, and medical organizations;
- Economic support of systemic enterprises, an extension of validity periods of permits for organizations, and deferral of enterprise tax payments [30,31,32].

We believe that other regions can use Moscow as an example of staged restriction removal because more than half of all positive COVID-19 tests in Russia were in Moscow due to the volume of testing and the number of cases. It should be noted that the time interval between the first and second stages of temporal restriction removal was seven days—one incubation period of disease development between the first and third stages of restriction removal (14 days)—which is two incubation periods [33].

By the recommendations of the Russian Federal State Agency for Health and Consumer Rights (Rospotrebnadzor), decisions to abolish (prolong) time limits at the regional level were necessary based on calculating the infection rate (Rt), calculated as the average number of people whom one patient infects before his or her isolation; the availability of free bed-space for the treatment of patients with COVID-19; and the test rate per 100 thousand population. As additional indicators to remove restrictions, it is recommended to use the level of lethality from COVID-19, reported weekly incidence of community-acquired pneumonia (total) compared to the average age level calculated over the past three years, and the proportion of persons with immunity to COVID-19 in the population according to the results of sample studies. Depending on the fulfillment of these criteria, the heads of regions made decisions on the staged lifting of restrictions [34].

Currently, during restrictive measures, the reprofiling of facilities to general hospitals for the needs of COVID-19 patients continues. In Moscow and other large cities in Russia, these reprofiled facilities are shopping centers or cinema halls [35].

Building rules and sanitary and epidemiological requirements for organizations carrying out medical activities establish the features of organizing infectious hospitals, which include planning, dividing “clean” and “dirty” zones, creating isolated ventilation, and the presence of chambers with access to the street [36,37].

Based on the survey results, we carried out a grouping and subsequent assessment of social and transport infrastructure facilities according to the risk of COVID-19 in three risk categories—high, medium, and low.

The healthcare system is faced with a high risk of nosocomial infection of medical workers and patients with COVID-19, leading to a shortage of medical personnel [38].

In the acute period of the pandemic, this problem was solved by intensifying work schedules, attracting non-core medical workers to work with infectious patients, and sending students of medical institutes and volunteers to hospitals.

After the end of the pandemic, some approaches to the content of basic training for doctors and nurses should be reviewed, increasing the number of hours on epidemiology and infectious diseases and introducing compulsory training in epidemiology and infectious diseases once every three years for all medical workers in continuing education programs [39].

We propose introducing the principle of “dual education” for training nurses based on specialties related to medicine, such as biology and veterinary medicine. In the case of possible epidemics and pandemics, it is advisable to involve nurses based on “biology” and “veterinary medicine” education to conduct diagnostic testing, maintain databases, and other auxiliary areas. Experienced nurses should be sent to provide medical care to patients.

We also offer an algorithm for the activities of governmental bodies, including administrative, diagnostic testing, vaccination, organizational, and medical personnel activities: (Table 4). This algorithm was used in Russia during 2020–2021. The stages of action determine the complication of the situation with the COVID-19 [40].

Russia has developed and begun to implement a set of governmental management measures to prevent the spread of emerging infections in the future, called “Sanitary Shield”. By Order of the Government of the Russian Federation dated 06.09.2021 No. 2461-r, a list of measures was approved to prevent the spread of newly emerging infections [41].

“Sanitary Shield” is a system of measures for preparing for future epidemics and their prevention. The main principle is to prevent new infections from entering Russia and minimize their penetration into the country. At the first stage, at the border’s sanitary and quarantine checkpoints, it is possible to test all those entering the country by express methods of diagnosing infections. These checkpoints will become modern laboratory complexes. At the second stage, a system of access to modern laboratory diagnostics will be created for all residents of Russia; stationary and mobile laboratory complexes will be created. At the third stage, modern laboratory complexes of high-level biosafety will be built in the regions of Russia that border foreign countries. This system will make the diagnosis of infections fast and accessible to everyone, anywhere in the country, and at any time, which will help laboratories decrypt any unknown infection in 24 h and develop a test system for any new infection in 4 days [42,43].

## 5. Conclusions

As part of implementing “Sanitary Shield”, the activities of Russian governmental bodies should be divided into decision-making groups to combat the coronavirus pandemic both currently and in the future. To ensure mobilization readiness for emerging epidemics and to create a reserve of medical personnel, the principle of “double” mobilization education has been proposed.

## Figures and Tables

**Table 1:** Hierarchy of informative features (risk criteria).

№	Risk Criterion	1-r	Rating
15	Social distancing	0.691358	16.5
16	Social distancing when visiting medical organizations	0.691358	16.5
13	Visiting polyclinics	0.678507	14.5
14	Hospital visits for patients with common illnesses (not COVID-19)	0.678507	14.5
12	Failure to comply with the mask regime in the workplace	0.594983	13
11	Traveling by public ground transport	0.583966	12
10	Visiting hairdressing salons, beauty salons	0.581808	11
7	Visiting grocery stores	0.524694	7.5
8	Visiting street outlets, kiosks	0.524694	7.5
5	Visiting pharmacies	0.503621	5.5
6	Visiting shops selling industrial and household goods	0.503621	5.5
3	Subway rides	0.487818	3.5
4	Trips in suburban electric trains (electric trains)	0.487818	3.5
1	Compliance with the mask regime in-ground public transport	0.324156	1.5
2	Compliance with the mask regime when visiting a store, pharmacy, etc.	0.324156	1.5

Low—orange; average—green; high—blue.

**Table 2:** Assessment in points by gradation of risk criteria (according to the respondents’ answers).

№	Risk Criterion	Gradation	Score	Average
16	Social distancing when visiting medical organizations	Yes	1	2.0
		No	3	
15	Social distancing	Yes	0.5	1.75
		No	3	
14	Hospital visits for patients with common illnesses (not COVID-19)	Yes	3.5	1.75
		No	0	
13	Visiting polyclinics	Yes	3.5	1.75
		No	0	
4	Trips in suburban electric trains (electric trains)	up to 1 h	0.5	1.75
		1–1.5 h	1	
		2 h or more	2	
11	Traveling by public ground transport	up to 1 h	0.5	1.75
		1–1.5 h	1	
		2 h or more	2	
3	Subway rides	up to 1 h	0.5	1.75
		1–1.5 h	1	
		2 h or more	2	
2	Compliance with the mask regime when visiting a store, pharmacy, etc.	Yes	0.5	1.25
		No	2	
1	Compliance with the mask regime in-ground public transport	Yes	0.5	1.25
		No	2	
8	Visiting street outlets, kiosks	Yes	1	0.5
		No	0	
7	Visiting grocery stores	Yes	1	0.5
		No	0	
10	Visiting hairdressing salons, beauty salons	Yes	1	0.5
		No	0	
6	Visiting shops selling industrial and household goods	Yes	1	0.5
		No	0	
5	Visiting pharmacies	Yes	1	0.5
		No	0	
12	Failure to comply with the mask regime in the workplace	Yes	1	0.5
		No	0	

Low—orange; average—green; high—blue.

**Table 3:** Classification of activities (professions) by risk of infection with COVID-19 (recipients of COVID-19).

№	Risk Categories	Infrastructure Facilities/Institutions	PPE Use
1	High	Medical institutions	
		Public transport facilities, including surface and underground (metro), suburban rail links	
		Homes for the elderly and disabled	
2	Average	Objects of aviation and railway transport	
		Food trade facilities	
		Pharmacy institutions	
2	Low	Office rooms	
		Objects of non-food trade	
		Public catering facilities	

**Table 4:** Step-by-step measures adopted by Russian governmental bodies to combat COVID-19.

N	Activities	Stages		
		Stage 1	Stage 2	Stage 3
1	Administrative	Use of masks and gloves by the population, disinfection of surfaces, disinfection of air in public buildings, social distancing	Elderly isolation, telecommuting, and learning	Lockdown: the population is at home, the termination of the work of enterprises, except for vital
2	Diagnostic testing	Development of diagnostic tests and their approbation	Test registration and industrial production, population testing	Mass testing of the population
3	Vaccination	Vaccine development and validation	Registration of vaccines, industrial production, the start of vaccination	Mass vaccination of the population
4	Organizational and medical	Construction of modular hospitals	Conversion of hospitals for infectious diseases, bed reserves	Organization of temporary hospitals (exhibition and concert halls)
5	Personnel	Training of GPs to work with infectious patients (reprofiling of specialists)	Attracting volunteers and medical students	Recruiting retired healthcare workers in hospitals

## Data Availability

The data presented in this study are available on request from the corresponding author.
